# DGAT: a dynamic graph attention neural network framework for EEG emotion recognition

**DOI:** 10.3389/fpsyt.2025.1633860

**Published:** 2025-07-21

**Authors:** Shihang Ding, Kaixuan Wang, Wenhao Jiang, Cong Xu, Hongjian Bo, Lin Ma, Haifeng Li

**Affiliations:** ^1^ Faculty of Computing, Harbin Institute of Technology, Harbin, China; ^2^ Shenzhen Academy of Aerospace Technology, Shenzhen, China

**Keywords:** EEG, emotion recognition, dynamic graph attention network, graph structure, affective computing

## Abstract

**Introduction:**

Emotion recognition based on electroencephalogram (EEG) signals has shown increasing application potential in fields such as brain-computer interfaces and affective computing. However, current graph neural network models rely on predefined fixed adjacency matrices during training, which imposes certain limitations on the model's adaptability and feature expressiveness.

**Methods:**

In this study, we propose a novel EEG emotion recognition framework known as the Dynamic Graph Attention Network (DGAT). This framework dynamically learns the relationships between different channels by utilizing dynamic adjacency matrices and a multi-head attention mechanism, allowing for the parallel computation of multiple attention heads. This approach reduces reliance on specific adjacency structures while enabling the model to learn information in different subspaces, significantly improving the accuracy of emotion recognition from EEG signals.

**Results:**

Experiments conducted on the EEG emotion datasets SEED and DEAP demonstrate that DGAT achieves higher emotion classification accuracy in both subject-dependent and subject-independent scenarios compared to other models. These results indicate that the proposed model effectively captures dynamic changes, thereby enhancing the accuracy and practicality of emotion recognition.

**Discussion:**

DGAT holds significant academic and practical value in the analysis of emotional EEG signals and applications related to other physiological signal data.

## 1 Introduction

Emotion is a response generated by the brain to various stimuli and represents a significant aspect of human intelligence (1). With the advancement of artificial intelligence technologies, emotion recognition techniques have emerged to enhance human-computer interaction efficiency by enabling computers to identify human emotions (2). Emotion recognition encompasses the identification of vocal tones, facial expressions, and physiological signals (3–5). Electroencephalogram (EEG) signals are electrical impulses produced by the brain during both resting states and when processing stimulus information. Due to the volume conduction effect of the brain, recorded EEG signals represent the overall response of the electrical activity of neuronal cells on the scalp (6). EEG signals possess high temporal resolution and can be easily collected, reflecting the brain’s processing of emotional stimuli, making EEG-based emotion recognition a focal point for researchers (7).

Graphs are widely employed for modeling complex relationships and structures in not only physiological signals and social networks (8), but complex biological systems from the omics to phenotypes (9–11). EEG signals, known for their real-time responsiveness (12), are more sensitive than peripheral neurophysiological signals to changes in emotional states, thereby revealing important features related to emotional states (13). In the context of EEG signals, graph neural networks (GNNs) utilize graph-structured data constructed from these signals as input to extract spatial structural features from EEG data, predicting labels based on the interactions between different electrodes (14). GNNs are effective in processing graph-structured data (15), capturing complex spatial relationships among electrodes while also modeling dynamic changes along the temporal dimension. Their application in EEG emotion classification has been gaining increasing attention (16). Researchers have explored the feasibility of emotion recognition and the decoding of emotional states and cognitive processes from EEG data using GNNs, given the advantages in EEG signal processing (17). (18) proposed a Dynamic Graph Convolutional Neural Network (DGCNN) that improves traditional graph convolutional networks by dynamically learning adaptive adjacency relationships among EEG electrodes, constructing graph adjacency matrices even under conditions of uncertain functional connectivity. The subject-dependent experiments on the SEED datasets achieved an accuracy of 90.4. (19) introduced a Graph-based Multi-task Self-Supervised Learning model (GMSS) for EEG emotion recognition, which captures intrinsic spatial relationships among different brain regions while exploring critical frequency bands for emotion recognition, achieving a maximum recognition accuracy of 86.37% on the SEED-IV dataset. (20) proposed a Regularized Graph Neural Network (RGNN) for EEG-based emotion recognition, leveraging the biological topological structures between different brain regions to capture both local and global relationships among EEG channels. Their approach was grounded in neuroscientific principles regarding the connectivity and sparsity of the adjacency matrix. Additionally, (21) introduced a Graph Embedded Convolutional Neural Network (GECNN) that combines local features from convolutional neural networks with global functional features to provide complementary emotional information, attaining a recognition accuracy of 92.93% on the SEED dataset.

Graph Attention Networks (GAT) adaptively assign weights to different nodes in the graph while capturing varying contributions from different brain regions under emotional states, thereby enhancing the model’s classification performance (22). Compared to traditional graph convolutional networks, GAT can learn correlation weights between each node and its neighboring nodes, offering a more flexible and efficient feature learning capability. These networks can reduce sensitivity to noisy signals through their attention mechanisms, automatically ignoring signal features irrelevant to emotion classification, thus improving model robustness (23). The attention mechanism of GAT facilitates understanding the contribution of channels to emotion classification tasks, contributing to explainability in neuroscience and further exploration of physiological mechanisms. (24) integrated a dual-branch attention module DBAM to enhance emotion recognition by combining channel and frequency information, but it mainly focuses on local features. However, the mechanism of emotion generation involves spatiotemporal relationships, and this dynamic process has not been fully reflected. Thus, although it improves the extraction of local features, it has certain limitations in understanding the overall dynamic response.3DCR-GAT (25) combines 3D convolution, residual networks, and graph attention to capture complex spatiotemporal dependencies as well as local features. However, despite its good performance in capturing spatiotemporal features, its core still revolves around modeling static features and it fails to fully leverage the advantages of dynamic adjacency structures, making it challenging to reflect emotional changes in real time.CR-GAT (26) attempts to enhance emotion recognition capabilities by learning sample-related graph representations, emphasizing the ability to construct multi-view representations. However, its focus on the dynamic evolution process of emotions remains insufficient. Additionally, it does not dynamically adjust feature structures, which may affect its sensitivity to changes in emotional states. LG-GAT (27) extracts task-related features through specific convolutional layers and combines local and global graph representations. However, it does not meticulously consider the temporal features of brain region activity, and the lack of dynamic feature capture may limit the overall performance of the model and its physiological interpretations.

Despite the progress made in the field of EEG-based emotion recognition, several shortcomings remain. EEG signals consist of data collected from multiple electrodes, which are spatially highly correlated and exhibit complex connectivity patterns between different brain regions. Additionally, the occurrence and evolution of emotions are closely linked to time and changes in brain functional areas. Current research methods predominantly focus on local features of EEG signals and do not fully exploit the valuable information contained within the topological structures of electrodes, making it challenging to capture the correlations among these signals. Furthermore, many existing studies often capture brain region activities statically, failing to consider the dynamic nature of emotional states over time. This dynamic evolution is crucial for understanding the mechanisms underlying emotion generation. However, current research lacks targeted designs addressing the interrelations among brain regions during emotional events and the dynamic evolution of emotions. Traditional static graph attention networks have inherent limitations, as they rely on predefined fixed adjacency matrices during training.

**Figure 1:**
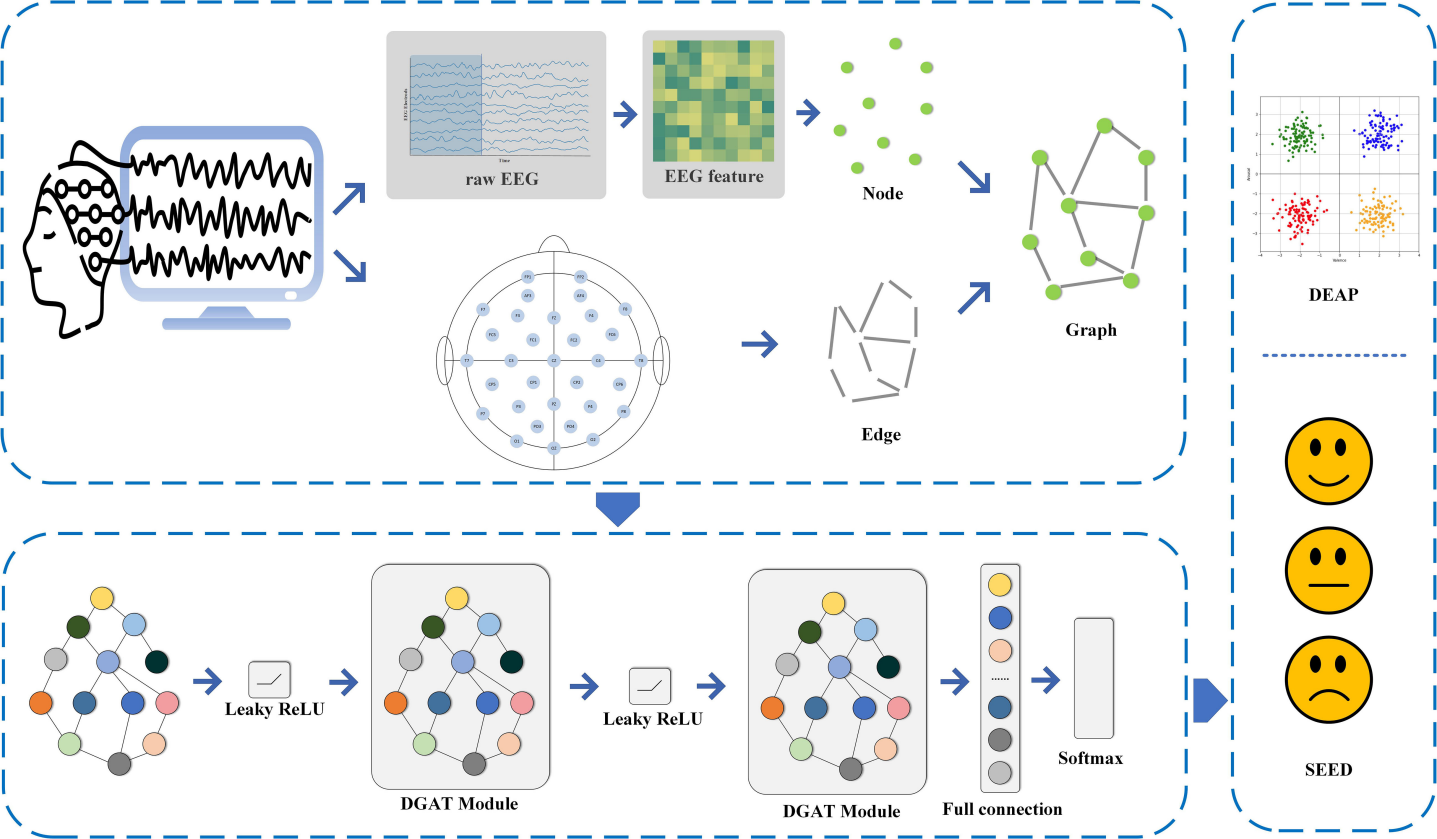
Overall working framework of the proposed model.

This dynamic modeling enables DGAT to effectively reflect the diversity and complexity involved in the emotion generation process. When constructing emotion recognition models, DGAT not only focuses on local signal features but also emphasizes the integration of global information. The model can better simulate the complex interactions between brain areas, reflecting the full spectrum of emotional states while deeply understanding the functions of various brain regions. The DGAT enhances the model’s robustness by automatically filtering out features that are irrelevant to emotion classification, thereby improving the overall reliability of the model. This adaptive characteristic is a significant advantage in emotion recognition, contributing to increased classification accuracy. DGAT not only aims to improve model performance but also offers stronger interpretability for emotion recognition. DGAT efficiently captures the dynamic evolution of emotional states, addressing how emotional conditions change over time. This dynamism is crucial for understanding the mechanisms of emotion, especially in decoding the interactions between brain regions and the neural mechanisms involved in the emotion generation process. To address the existing challenges in the field of EEG emotion recognition, this paper proposes a method for emotion recognition based on a Dynamic Graph Attention Network (DGAT). The primary contributions of this paper are as follows:

We introduce a DGAT framework for EEG emotion recognition that improves the accuracy of emotion detection by dynamically learning the relationships between different channels. We design a dynamic attention mechanism tailored for EEG emotion recognition, enhancing the expressive capability of features and providing a nuanced understanding of emotional states. Through dynamic relational learning, DGAT reduces reliance on specific adjacency structures. The overall framework of the proposed model is illustrated in **Figure 1**. We conduct comprehensive and rigorous comparative experiments between the proposed method and several representative baseline models across different datasets, effectively demonstrating the advancements of the proposed model. The remainder of the paper is organized as follows: The Materials and Methods section introduces the datasets utilized and the proposed methodology. The Results section presents the experimental outcomes obtained and comparisons with existing methods. The Discussion section interprets and discusses the implications of the results.

## 2 Materials and methods

In this section, we mainly introduce the overview of the dataset, the preprocessing process, the graph data construction, and the specific details of the proposed model.

### 2.1 Emotion database

**Figure 2:**
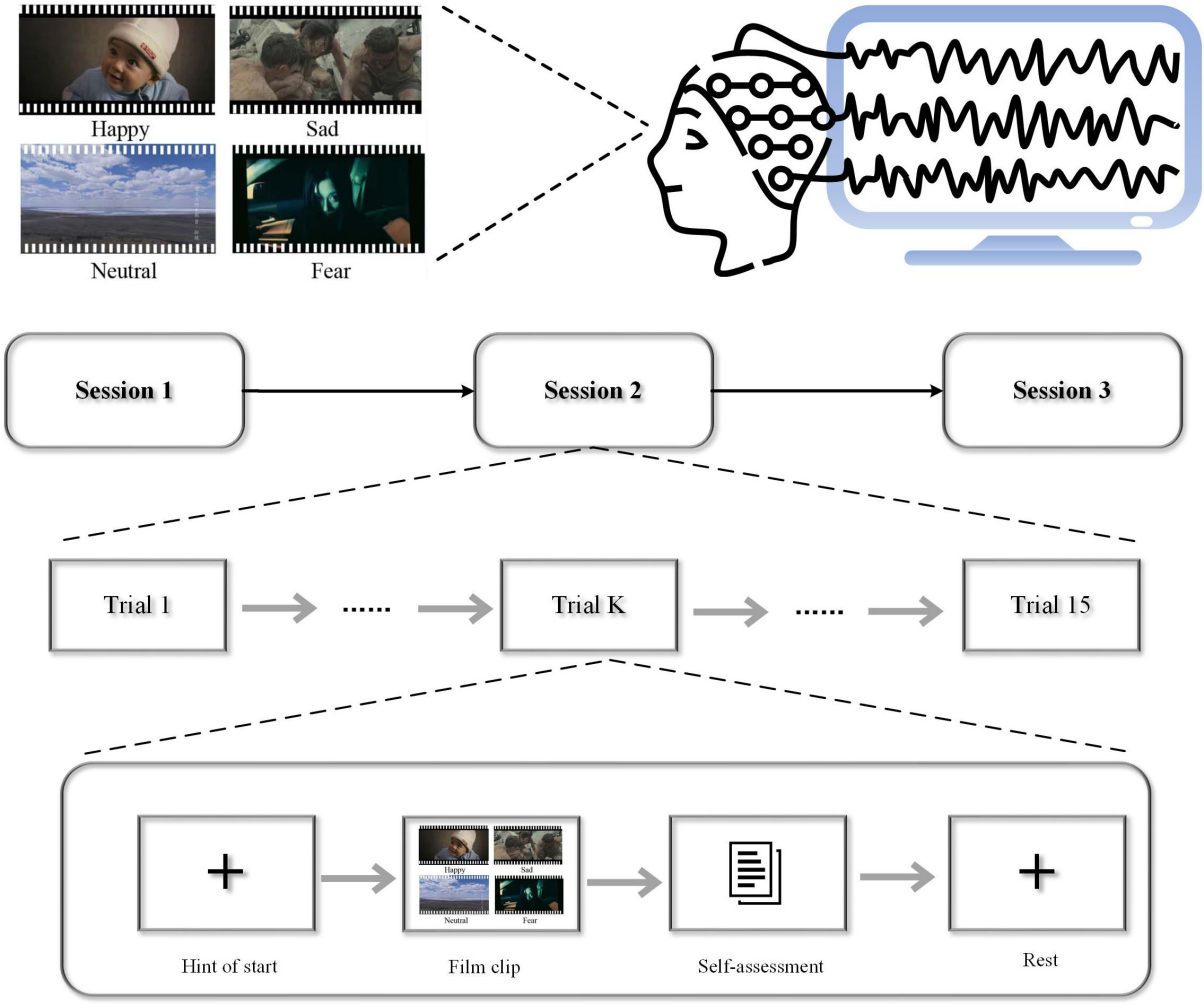
Schematic diagram of the experimental process for the SEED emotion EEG dataset.

**Figure 3:**
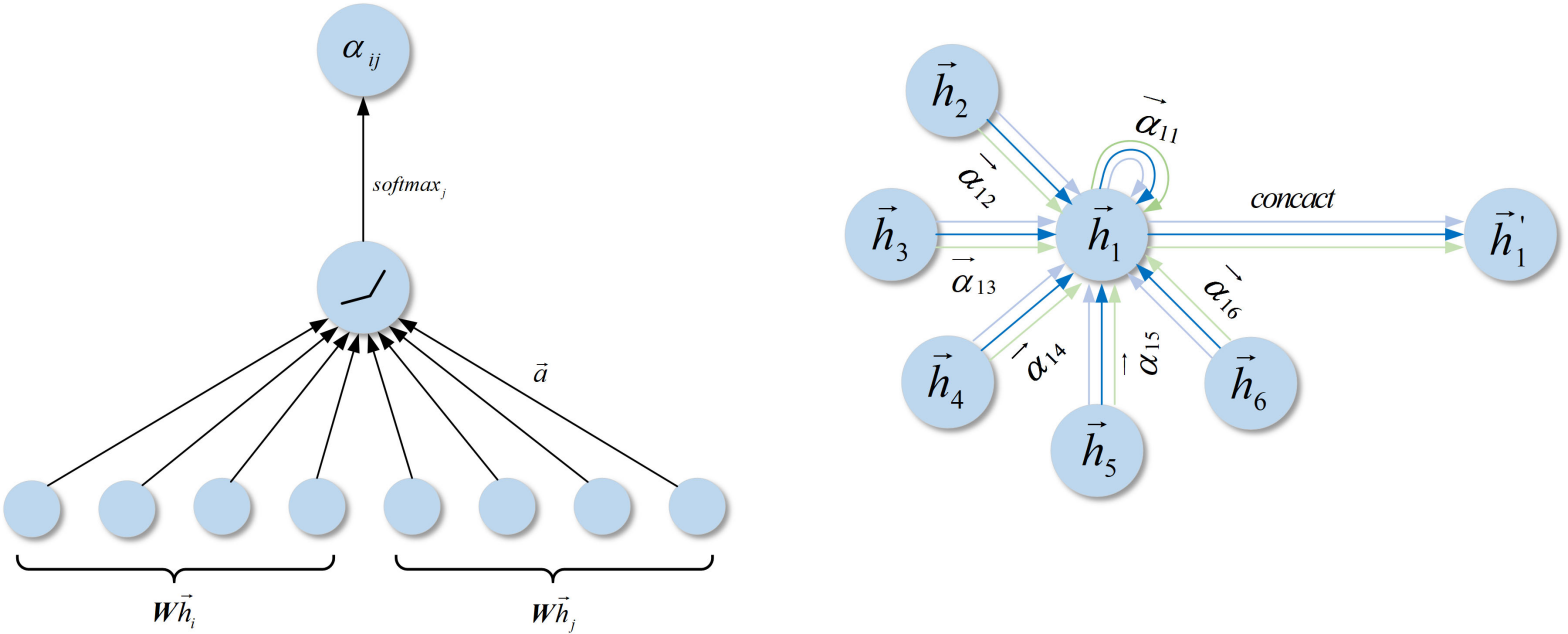
Left: The attention mechanism $a\left(W{\vec{h}}_{i},W{\vec{h}}_{j}\right)$ is parameterized by the weight vector. Right: Schematic representation of multi-head attention (with k=2 ) on nodes within their neighborhoods, where arrows of different colors indicate independent attention computations. The computation method of ${\vec{h}}_{1}^{\prime }$ involves concatenating the aggregated features from each attention head.

The SEED (SJTU Emotion EEG Dataset) is an electrophysiological dataset collected by Shanghai Jiao Tong University (28). The experiment utilized emotional films to elicit emotions in participants. They comprise a combination of both video and audio channels, providing participants with real-life scenarios that can induce more intense emotions and psychological changes. The dataset consists of stimuli selected from 15 different emotional video clips, each approximately four minutes in length. It includes videos representing three distinct emotions (positive, neutral, and negative), with five video segments for each emotion. The experiment involved a total of 15 participants (8 females and 7 males). EEG signals were continuously recorded using a 64-channel EEG system (Neuroscan system) at a sampling rate of 1000 Hz, following the standard 10–20 system. As illustrated in **Figure 2**, the experiment consists of 15 trials. Each session includes a 5-second introductory segment, a 4-minute emotional video, a 45-second self-assessment, and a 15-second rest period. **Figure 3** depicts a schematic diagram of the emotional EEG data experimental process.

**Figure 4:**
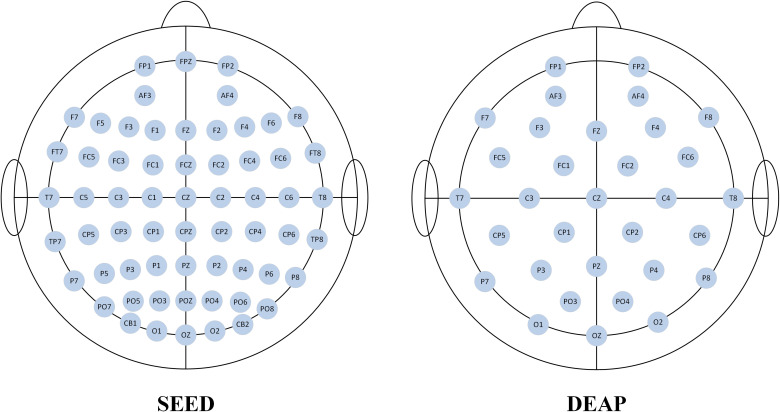
Electrode position distribution maps for the two datasets.

DEAP (Database for Emotion Analysis using Physiological Signals) is a multimodal physiological signal dataset collected through cognitive experiments for emotional research (29). This dataset utilizes music video materials to elicit emotions in participants. The experiment involved data from 32 participants, comprising 16 males and 16 females, which included physiological signals such as EEG and EOG. EEG signals were collected using a standard 10–20 system with a 32-channel EEG acquisition system at a sampling rate of 512 Hz. **Figure 4** illustrates the electrode position distribution for the two datasets used in this study. During the experiment, participants were asked to watch 40 music videos, each lasting 1 minute and featuring different emotional content. Each data segment lasted 63 seconds, which included 3 seconds of baseline data prior to the formal experiment and 60 seconds of music video viewing data. The details of the two datasets are shown in **Table 1**.

**Table 1:** Overall parameters of the datasets.

3 Parameter	DEAP	SEED
Subjects	32	15
Trials/Film clips	40	15
Each clip duration	1-min	4-min
Sessions/experiments	1	3
EEG electrodes	32	62
Sampling rate	512 Hz	200 Hz
Emotion category	4-class	3 class

### 2.2 Data pre-processing

For the DEAP dataset, we followed the same preprocessing steps as outlined in (30). The first 3 seconds of baseline data from the original EEG signals were removed, and the signals were subsequently downsampled to 128 Hz. Eye movement noise was eliminated using the noise removal techniques described in (29). A bandpass filter with a range of 4–45 Hz was applied to remove low-frequency and high-frequency noise. The EEG signals were then segregated into four frequency bands: θ (4–8 Hz), α (8–13 Hz), β (13–30 Hz), and γ (30–45 Hz). Differential entropy (DE) features were extracted from the four frequency bands, employing a short-time Fourier transform with a 4-second non-overlapping Hanning window.

For the SEED dataset, we followed the same preprocessing steps as described in (28). The original EEG signals were downsampled to 200 Hz. A bandpass filter with a frequency range of 1–70 Hz was utilized to obtain the desired frequency range and eliminate power line noise. During the EEG acquisition process, eye movement artifacts may interfere with the data; hence, independent component analysis (ICA) was employed to remove these artifacts. The EEG signals were divided into five frequency bands: δ (1–4 Hz), θ (4–8 Hz), α (8–14 Hz), β (14–31 Hz), and γ (31–50 Hz). Differential entropy (DE) features were extracted from the five frequency bands using a short-time Fourier transform with a 4-second non-overlapping Hanning window. Consequently, the final dimensionality of the features extracted from the 62-channel EEG data amounted to 62×5 = 310 dimensions.

### 2.3 Graph attention block

Graph neural networks (GNNs) can aggregate information between different nodes into high-dimensional representations, thereby enabling effective representation of EEG signals and achieving accurate classification of emotion-related EEG signals. A graph can be defined as G={V,E,W} , where $V=\left\{v_{1},v_{2},\ldots ,v_{N}\right\}\in R^{N}$ denotes the set of nodes containing N nodes, and $E=\left[e_{jk},j=1,2,\ldots ,N,k=1,2,\ldots ,N\right]$ represents the set of edges connecting these nodes. The adjacency matrix $W\in {\mathbb{R}}^{N\times N}$ is utilized to describe the connectivity relationship between any two nodes, with the element W representing the connection between node i and node j . GNNs take node features and the adjacency matrix as input, where the node features are extracted from the raw signals, and the adjacency matrix captures the relationships among the nodes. The traditional method for calculating the elements of the adjacency matrix is given by Equation 1.


(1) $$w_{ij}=\{\begin{array}{c} \exp\left(-\frac{{\left[dist(i,j)\right]}^{2}}{2\theta^{2}}\right),if\;dist(i,j)\leq \tau \;\\ 0,otherwise \end{array}$$

where τ and θ are two fixed parameters, dist(i,j) represent the distance between node i and node j .

For a given graph-structured data, the input to the graph neural network consists of features of different graph nodes $h=\left\{{\vec{h}}_{1},{\vec{h}}_{2},\ldots ,{\vec{h}}_{N}\right\},{\vec{h}}_{i}\in {\mathbb{R}}^{F}$ , where N represents the number of nodes and F denotes the dimensionality of the input features for each node. After learning through the network, the output features are denoted as $h^{\prime }=\left\{{\vec{h}}_{1}^{\prime },{\vec{h}}_{2}^{\prime },\ldots ,{\vec{h}}_{N}^{\prime }\right\},{\vec{h}}_{i}^{\prime }\in {\mathbb{R}}^{F^{\prime }}$ . It is noteworthy that the dimensionality of the output features $F^{'}$ may differ from that of the input features F .

A shared weight matrix $W\in {\mathbb{R}}^{F^{\prime }\times F}$ is applied across all nodes, which is learned simultaneously during model training using the backpropagation algorithm. The importance of each node’s features is calculated pairwise, $e_{ij}$ indicating the significance of node i to node j in Equation 2. In this calculation, only the node itself and the first-order neighborhood nodes are considered.


(2) $$e_{ij}=a\left(W{\vec{h}}_{i},W{\vec{h}}_{j}\right)$$

To facilitate the comparison of attention parameters among different nodes, the softmax function is applied to normalize the attention parameters of node j with respect to other nodes in Equation 3.


(3) $$\alpha_{ij}=softmax_{j}\left(e_{ij}\right)=\frac{\exp\left(e_{ij}\right)}{\sum_{k\in N_{i}}\exp\left(e_{ik}\right)}$$

The calculation formula for the attention parameters in the graph attention network is given in Equation 4, where $\cdot^{T}$ denotes the matrix transpose operation and ‖ represents the matrix concatenation operation, with LeakyReLU utilized as the activation function. The computation process of the attention mechanism is illustrated in **Figure 4**.


(4) $$\alpha_{ij}=\frac{\exp\left(LeakyReLU\left({\vec{a}}^{T}\left[W{\vec{h}}_{i}\|W{\vec{h}}_{j}\right]\right)\right)}{\sum_{k\in N_{i}}\exp\left(LeakyReLU\left({\vec{a}}^{T}\left[W{\vec{h}}_{i}\|W{\vec{h}}_{k}\right]\right)\right)}$$

Applying the attention parameters to the feature vectors corresponding to the nodes yields the output in Equation 5:


(5) $${\vec{h}}_{i}^{\prime }=\sigma \left(\sum_{j\in N_{i}}\alpha_{ij}W{\vec{h}}_{j}\right)$$

By employing a multi-head attention mechanism, different heads can capture diverse information. The final fusion of information from multiple heads enhances the stability of the self-attention learning process. The ultimate output for each node is given by Equation 6:


(6) $${\vec{h}}_{i}^{\prime }={\|}_{k=1}^{K}\sigma \left(\sum_{j\in N_{i}}\alpha_{ij}^{k}W^{k}{\vec{h}}_{j}\right)$$

Let K denote the number of attention heads, $\alpha_{i,j}^{k}$ represent the normalized self-attention parameters learned by the kth attention head, and $W^{k}$ denote the weight matrix corresponding to the kth attention head.

### 2.4 Dynamic graph attention block

**Figure 5:**
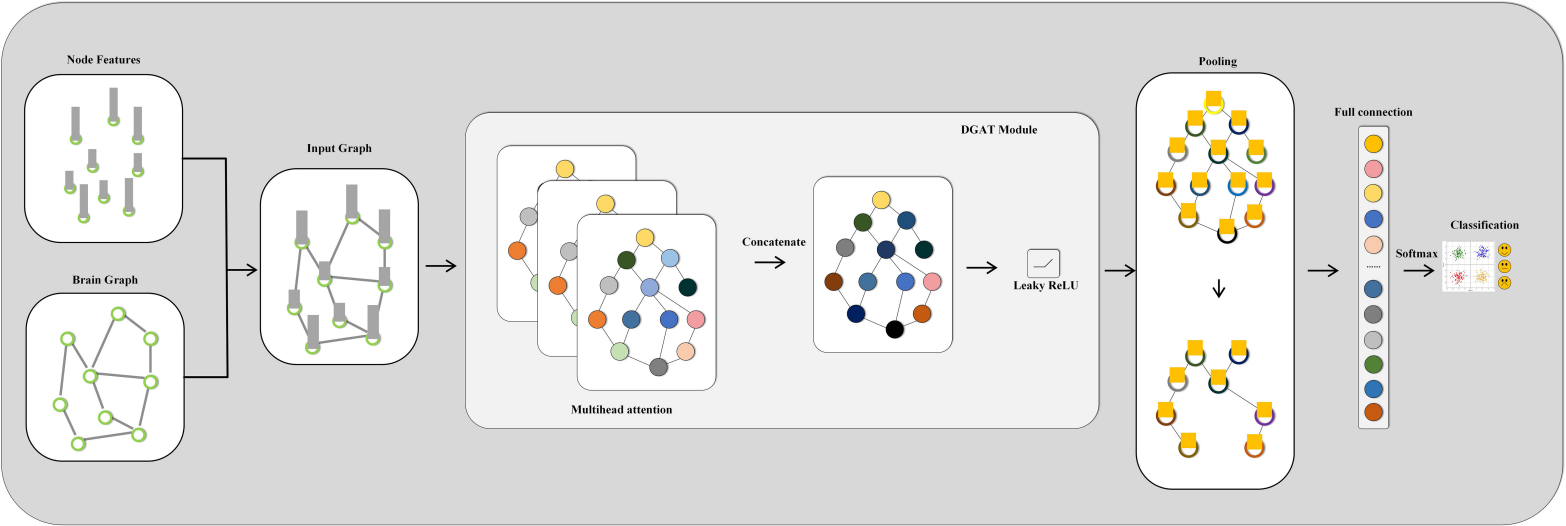
Schematic diagram of the network architecture.


The diagram of the proposed network architecture is illustrated in **Figure 5**. In the dynamic graph attention network, the adjacency matrix from the static graph attention network serves as a trainable and learnable parameter. This enables the comprehensive learning of relationships between different EEG channels through a dynamic adjacency matrix. During the learning process, the edge connection weight between node i and node j can be computed in Equation 7:

(7) $$e_{ij}=a\left(W{\vec{h}}_{i},W{\vec{h}}_{j}\right)$$

By combining the initialized adjacency matrix with the edge connection weights obtained from the Dynamic Graph Attention Network through the attention mechanism, we obtain the following formula Equation 8, where β represents the momentum parameter:


(8) $$w_{ij}\leftarrow \beta w_{ij}+(1-\beta )e_{ij}$$

The updated attention parameters of node j with respect to other nodes are normalized using the softmax function as Equation 9.


(9) $$\alpha_{ij}=softmax_{j}\left(e_{ij}\right)=\frac{\exp\left(d_{ij}\right)}{\sum_{k\in N_{i}}\exp\left(d_{ik}\right)}$$

The attention parameters are applied to the feature vectors corresponding to the graph nodes, resulting in Equation 10:


(10) $${\vec{h}}_{i}^{\prime }=\sigma \left(\sum_{j\in N_{i}}\alpha_{ij}W{\vec{h}}_{j}\right)$$

### 2.5 Feature extraction

Researchers have confirmed that differential entropy (DE) features can serve as effective attributes for the classification and recognition of EEG-based emotions. Therefore, we extracted DE features from the original EEG signals for further classification. Differential entropy is an extension of Shannon entropy for continuous variables, quantifying the overall uncertainty of the probability distribution of continuous random variables and reflecting the frequency variation characteristics of EEG signals. EEG signals over shorter time intervals can be approximated as Gaussian distributions $N\left(\mu ,\sigma_{i}^{2}\right)$ , and thus can be considered to be characterized by a Gaussian probability density function. The calculation of DE features from EEG signals approximates them as the logarithm of the power spectral density defined within a specified frequency range. The mathematical expression of this calculation is in Equation 11:


(11) $$\begin{array}{l} DE=-\int_{a}^{b}f(x)\log(f(x))dx\\ \;=-\int_{a}^{b}\frac{1}{\sqrt{2\pi \sigma^{2}}}\exp\frac{{\left(x-\mu \right)}^{2}}{2\sigma^{2}}\log\left(\frac{1}{\sqrt{2\pi \sigma^{2}}}\exp\frac{{\left(x-\mu \right)}^{2}}{2\sigma^{2}}\right)dx\\ \;=\frac{1}{2}\log\left(2\pi e\sigma^{2}\right) \end{array}$$

Let x denote the input EEG signal, μ represent the mean of the signal over a specified time range, σ denote the variance, and a and b signify the time range of the signal. The differential entropy features are calculated for EEG signal data across all frequency bands, resulting in $X=\left[X_{1},X_{2},\ldots ,X_{T}\right]\in {\mathbb{R}}^{T\times C\times B}$ , where T represents the length of the differential entropy features, C denotes the number of EEG electrodes, and B signifies the number of frequency bands.

### 2.6 Experimental settings

The performance of the proposed method was tested using both subject-dependent and subject-independent experimental frameworks. In the subject-dependent experiments with the DEAP dataset, three types of tasks were conducted: binary classification tasks for arousal (HA/HV and LA/LV) and a four-class classification task for valence-arousal (HAHV, HALV, LAHV, and LALV). The DEAP dataset was subjected to within-subject experiments utilizing five-fold cross-validation, with 80% of the data allocated for training and the remaining 20% for testing. For the SEED dataset, a three-class classification experiment was carried out, categorizing the signals into positive, neutral, and negative classes, also utilizing subject-dependent experiments with five-fold cross-validation. In the subject-independent experiments, each subject was alternately treated as the test set while the data from the other subjects served as the training set to assess the performance of the proposed method.

All experiments were conducted under identical software and hardware conditions, including dataset partitioning and parameter settings. The model was implemented on a Dell desktop computer equipped with an Intel Core i5–13400 processor (2.50 GHz) and an Nvidia GeForce RTX 3060 Ti GPU. The software environment consisted of the Windows 10 operating system, the Python 3.9 programming language, and the PyTorch 1.10.1 deep learning framework.

During model training, the obtained feature vectors are passed through a full connection layer to perform dimensionality reduction, subsequently generating prediction labels to achieve the final classification results. To evaluate the classification outcomes of the model, we use accuracy as the performance metric, as shown below:


(12) $$Accuracy=\frac{\left(TP+TN\right)}{\left(TP+TN+FP+FN\right)}$$

This formula is an example for binary classification tasks. The total sample size is the sum of true positive (TP) results, true negative (TN) results, false positive (FP) results, and false negative (FN) results, while the numerator represents the total of TP and TN, which indicates the number of correctly predicted samples.

For the proposed model, the final loss function is defined by minimizing the cross-entropy and the L2 regularization term, with Adam selected as the optimizer. The momentum parameter is set to the default value of 0.5. During the training process, the learning rate and batch size are set to 0.001 and 64, respectively.

## 3 Results

### 3.1 Emotion recognition results

**Table 2** displays the performance of the proposed DGAT framework in the subject-dependent emotion recognition task on the SEED dataset, achieving an accuracy of 94.00% (with standard deviation of 2.60%) for the classification of positive, neutral, and negative emotions. In the subject-independent experiments, we obtained an average accuracy of 90.03%(with standard deviation of 4.28%). As shown in **Table 2**, these results represent the best performance achieved to date on the SEED dataset, demonstrating that the dynamic graph attention network can enhance the accuracy of emotion recognition from EEG signals by leveraging dynamic adjacency matrices and multi-head attention mechanisms.

**Table 2:** Comparison of average accuracy and standard deviation for subject-dependent and subject-independent emotion recognition experiments on the SEED dataset.

Method	Accuracy (mean/std)	
	Subject-dependent	Subject-independent
SVM	83.99/09.72	72.62/10.38
DBN[28]	86.08/08.34	76.14/09.64
DGCNN[18]	90.40/08.49	84.91/07.71
BiHDM[31]	91.01/08.91	85.40/07.53
BiDANN[30]	92.38/07.04	84.14/06.87
DGAT	**94.00/02.60**	**90.03/04.28**

The bold value means the best performance.

The experimental results on the DEAP dataset are presented in **Table 3**. In the subject-dependent experiments, the proposed DGAT model achieved an average accuracy of 93.55% (with standard deviation of 3.89%) for the dimension of valence, and an average accuracy of 93.19% (with standard deviation of 3.65%) for the dimension of arousal. For the subject-independent experiments, our proposed model attained an average accuracy of 84.27% (with standard deviation of 13.74%) for the dimension of valence, and an average accuracy of 83.84% (with standard deviation of 10.20%) for the dimension of arousal, as shown in **Table 4**. The results indicate that the DGAT model achieved optimal performance.

**Table 3:** Comparison of average accuracy and standard deviation for subject-dependent emotion recognition experiments on the DEAP dataset for valence and arousal dimensions.

Method	Accuracy (mean/std)	
	Valence	Arousal
DCCA[32]	85.62/03.48	84.33/02.25
ATDD-LSTM[33]	90.87/11.32	90.91/12.95
DBCN[34]	90.93/03.90	89.67/04.50
SFE-Net[35]	92.49/-	91.94/-
DGAT	**93.55/03.89**	**93.19/03.65**

The bold value means the best performance.

**Table 4:** Comparison of average accuracy and standard deviation for subject-independent emotion recognition experiments on the DEAP dataset for valence and arousal dimensions.

Method	Accuracy (mean/std)	
	Valence	Arousal
LCAA-Net[36]	65.90/09.50	69.50/9.70
FLDNet[37]	83.85/11.34	78.22/10.14
ATDD-LSTM[33]	72.97/06.57	69.06/06.37
DBCN[34]	83.98/13.20	79.45/10.60
DGAT	**84.27/**13.74	**83.84/10.20**

The bold value means the best performance.

**Figure 6:**
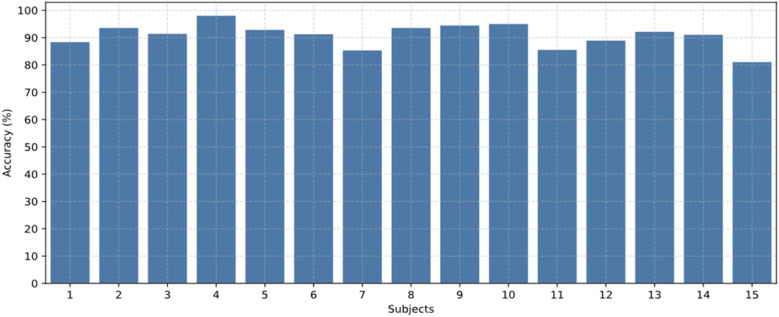
Comparison of emotion recognition results for all participants in the SEED dataset.

**Figure 7:**
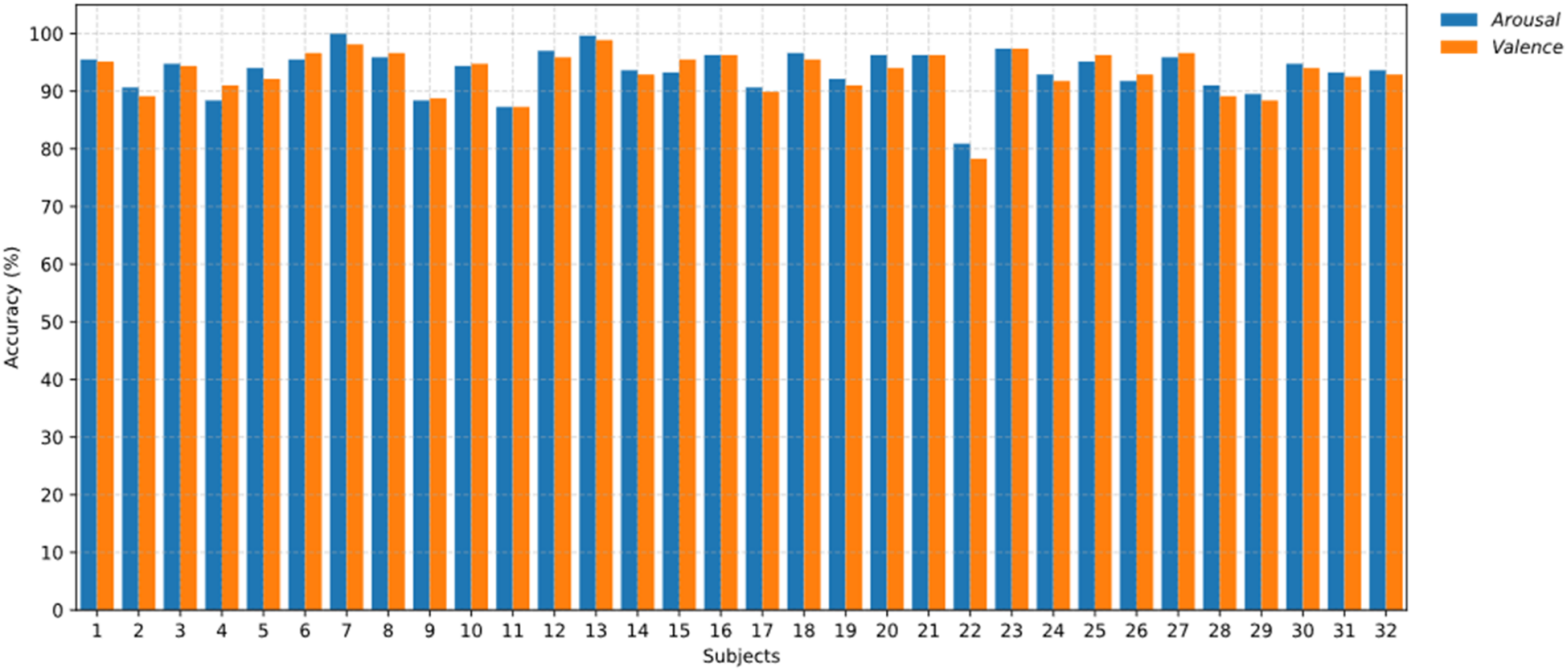
Comparison of valence and arousal recognition results for all participants in the DEAP dataset.

**Figure 6** presents the results of the subject-dependent emotion recognition accuracy for the 15 participants included in the SEED dataset. **Figure 7** displays the recognition accuracy results for the subject-dependent experiments involving 32 participants in the DEAP dataset. Among the 32 participants, only Participant 22 exhibited lower accuracy, while the recognition accuracies for valence and arousal dimensions remained consistently high for the other participants.

### 3.2 Additional analysis and results

**Figure 8:**
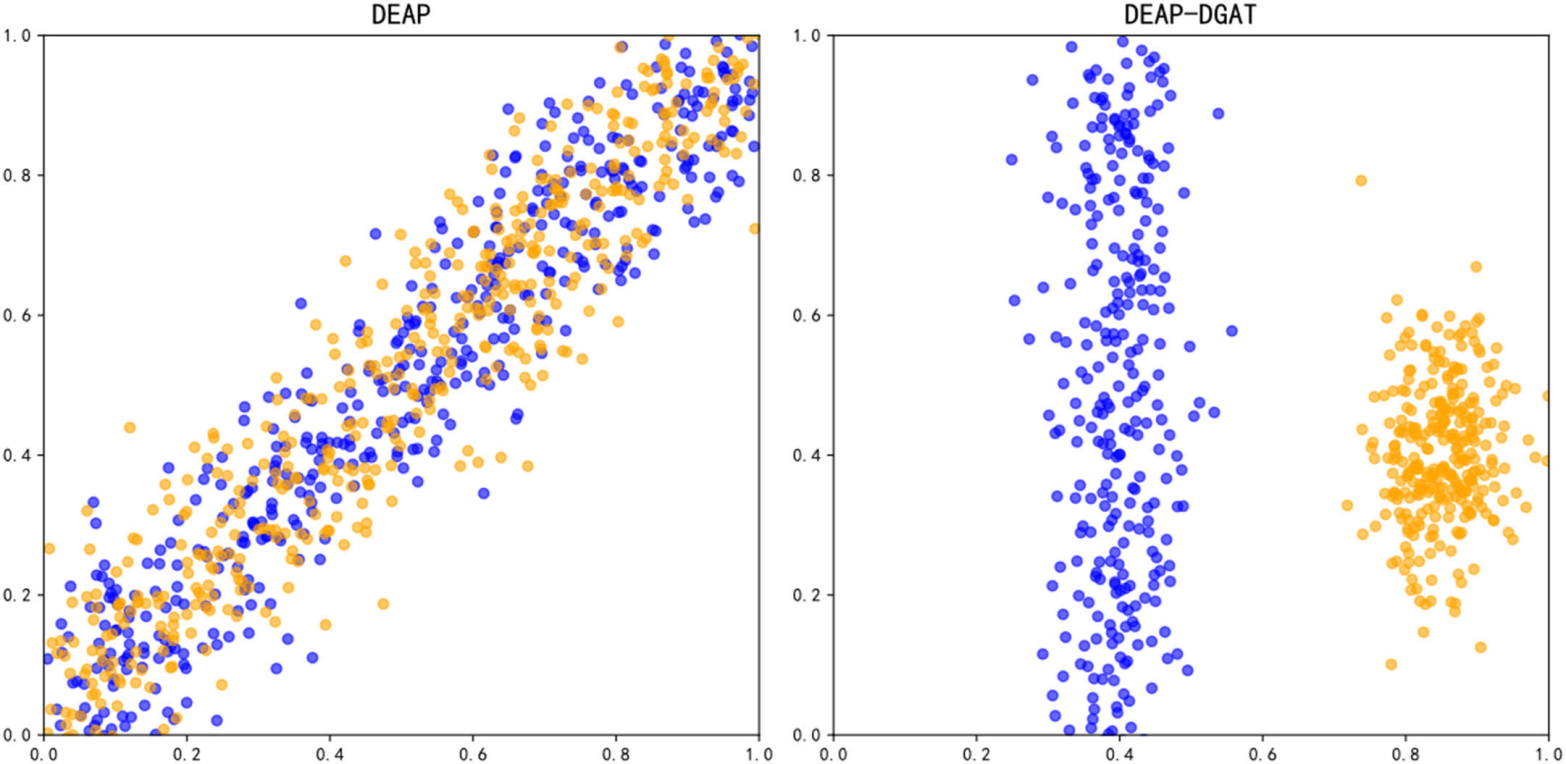
Feature visualization before and after training on the DEAP dataset.

To demonstrate the effectiveness of DGAT in extracting high-level abstract features, we performed a t-SNE visualization analysis using DE features from a portion of the dataset based on experimental results, as shown in **Figure 8**. It is evident that after training with DGAT, the sample distribution becomes clearer, and the degree of sample confusion is reduced. This confirms the effectiveness of the proposed DGAT in extracting features related to emotional states.

**Figure 9:**
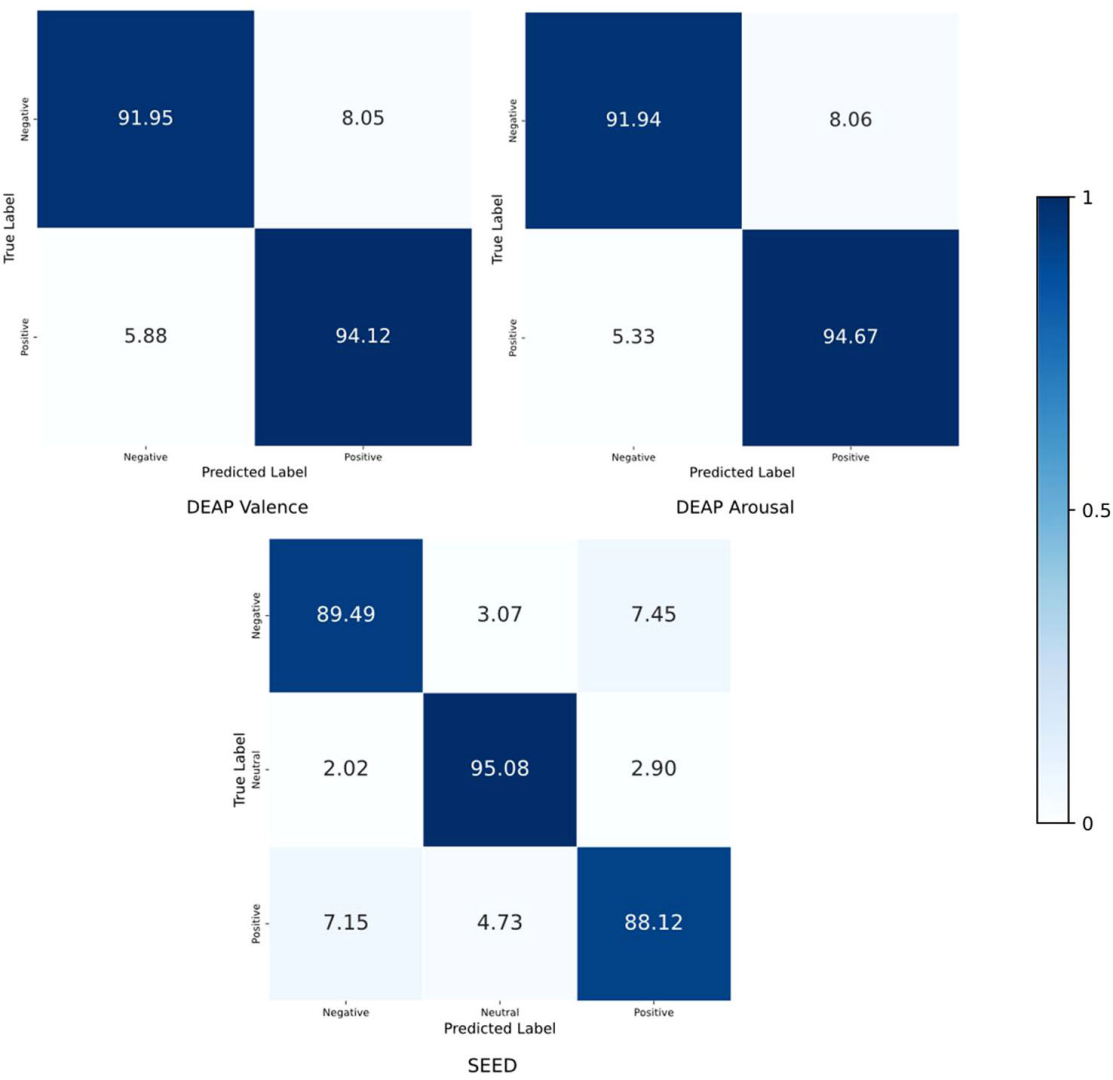
Confusion matrix of results from the SEED and DEAP datasets.

To intuitively display the prediction results for each emotional category across different datasets while also comparing the predicted results of the classification model with the true labels for a deeper understanding of the model’s performance, we computed the confusion matrix for the proposed DGAT model, as shown in **Figure 9**. In the confusion matrix, the sum of the elements in each row represents the total number of samples, the diagonal elements indicate the percentage of samples correctly classified for each emotion, and the remaining elements represent the percentage of samples that were misclassified.

**Figure 10:**
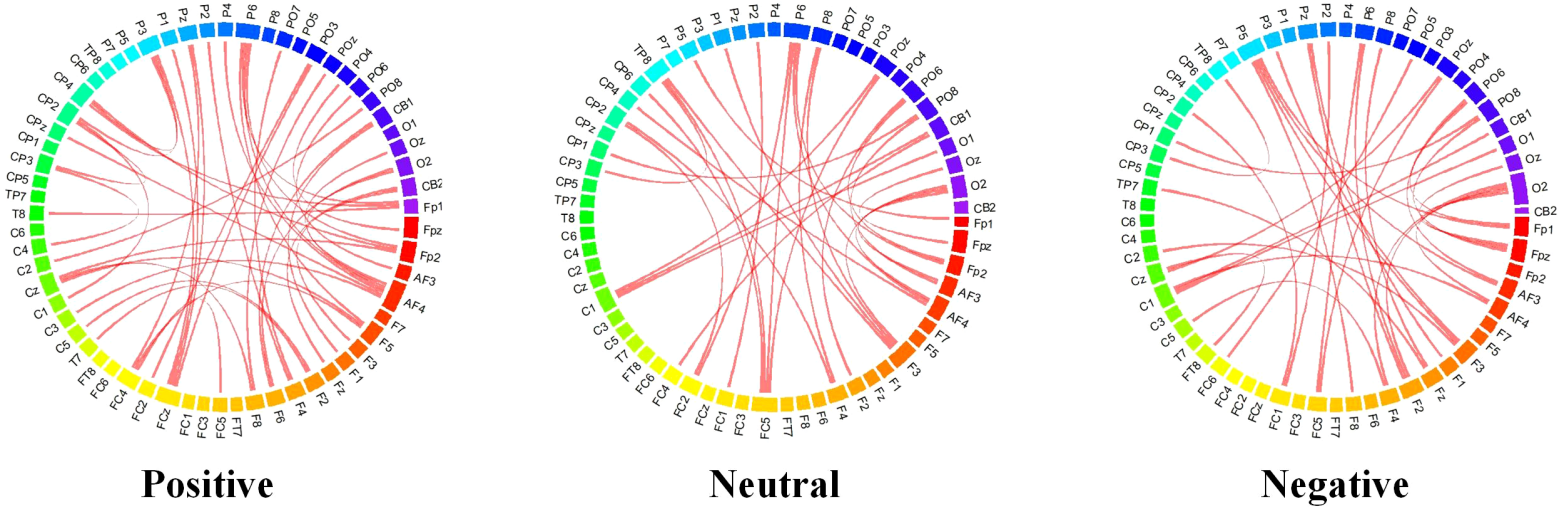
Comparison of connection for different emotional states.

**Figure 11:**
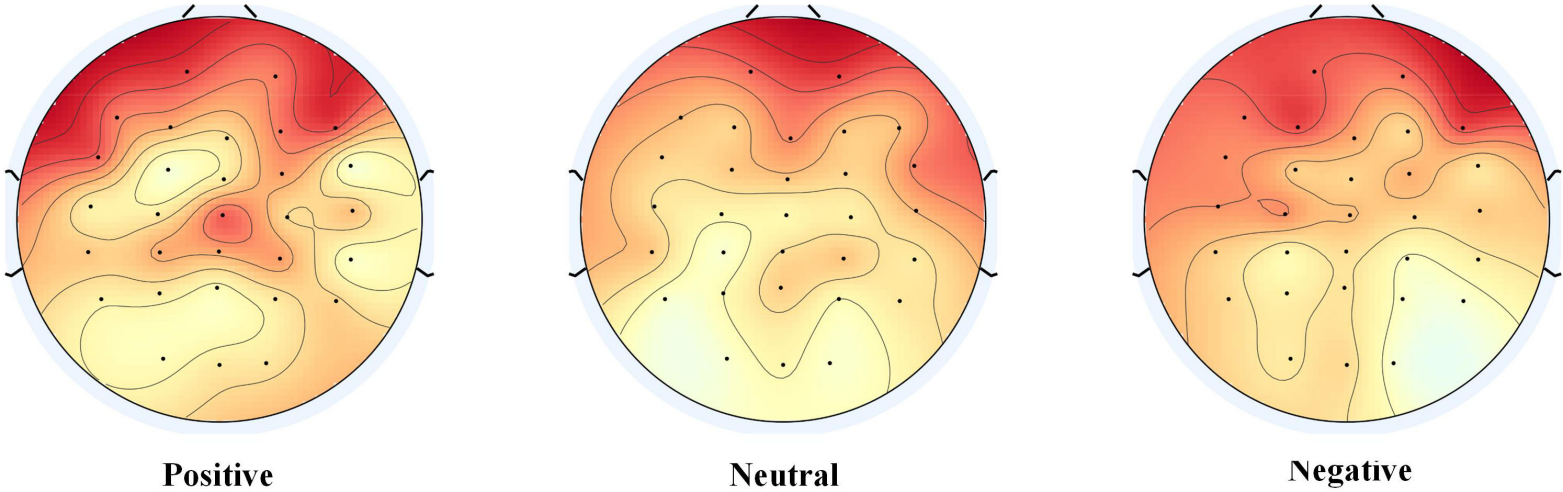
Comparison of attention weight distribution for different emotional states.

In order to compare the differences in the connections learned by the models during different emotional processing and to further explore the relationship between connections and emotional pathways, we calculated the connection weights after model training for three emotional states, following the methods outlined in the authoritative literature (38). The experimental results are shown in **Figure 10**. It can be observed that the connectivity of brain regions related to positive emotions is more active, exhibiting stronger connections, while in negative emotional states, the connectivity of the frontal lobes is enhanced. This reveals the network connectivity of the brain under different emotional states. To compare the distribution of attention weights across different emotional states, we referenced the relevant research methods in the authoritative literature (39) and plotted a topographic map of the brain based on the electrode distribution, as shown in **Figure 11**. The emotion states are highly correlated with brain regions, with attention weights primarily concentrated in the frontal and temporal lobes. This reveals the relationship between attention distribution and the associated brain regions, providing an effective basis for interpreting the model results.

**Figure 12:**
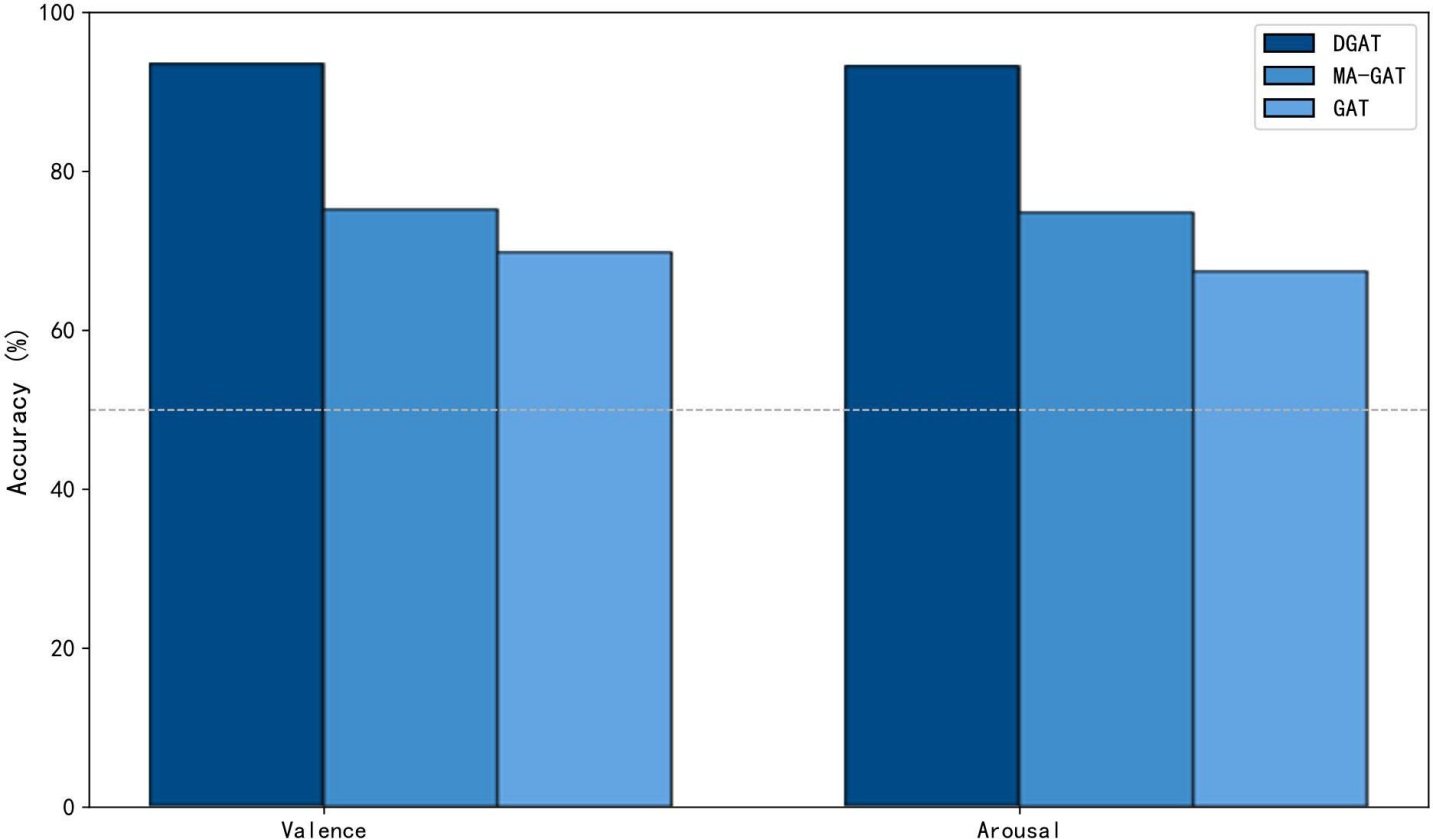
Results of the ablation experiment for the DGAT model.

To validate the effectiveness of the proposed DGAT model, we conducted an ablation experiment by replacing DGAT with a GAT that uses a fixed adjacency matrix and multi-head attention mechanism. In addition, we conducted a sensitivity test on the number of attention heads. The experimental results are shown in **Figure 12** and **Table 5**. It can be observed that after replacing the DGAT module with GAT, the model’s emotional recognition performance significantly decreases. This evidence supports that DGAT can dynamically learn the relationships between different channels, utilizing a dynamic adjacency matrix to reduce reliance on specific adjacency structures, thereby enhancing the accuracy of emotion recognition from EEG signals.

**Table 5:** Comparison of experimental results with different numbers of attention heads.

Number of Head	Accuracy (mean/std)
1	89.39/04.97
2	92.78/04.31
3	**94.00/03.89**

The bold value means the best performance.

## 4 Discussion

We present a dynamic graph attention network-based framework for EEG emotion recognition, referred to as DGAT. Experiment results on the SEED and DEAP emotional EEG datasets demonstrate that our proposed model outperforms existing methods and achieves state-of-the-art results in both subject-dependent and subject-independent experiments. For the SEED dataset, we extracted differential entropy features from the raw EEG signals and further constructed a graph structure by integrating these features with EEG electrodes as input to the dynamic graph attention network. This approach enables the extraction of higher-level graph structural information, effectively capturing the relationships among different electrodes. Additionally, we employed a multi-head attention mechanism to aggregate information using various attention weights, thereby enhancing classification accuracy. In the three-class subject-dependent emotion recognition task (positive, neutral, and negative) and in the subject-independent tasks, our proposed model consistently achieved higher accuracy than previous state-of-the-art (SOTA) models. This indicates that the proposed method is more robust for cross-subject emotion recognition, making it more suitable for applications in emotion-based brain-computer interfaces. In the DEAP dataset, we conducted binary classification experiments on valence and arousal based on subject-dependent and subject-independent tasks to validate the effectiveness of the proposed model. The results indicated that our model achieved the highest recognition performance across these tasks. Among 32 subjects, only Subject 22 exhibited a lower accuracy, while the recognition accuracy for valence and arousal remained high for the other subjects. Furthermore, the accuracy for valence recognition was higher than that for arousal recognition, which is consistent with previous research findings. The observed discrepancies across subjects’ data samples and inherent noise in the EEG data contribute to this phenomenon.

The brain’s processing of emotions mainly focuses on the frontal and parietal lobes, a finding that is consistent with results from studies based on functional magnetic resonance imaging (fMRI) (40). The results indicate that the connectivity of brain regions associated with positive emotions is more active, displaying stronger connections. This suggests that when individuals experience feelings of happiness or fulfillment, communication and collaboration between the relevant brain regions occur more frequently, thereby enhancing the intensity and clarity of the emotional experience. In contrast, under negative emotional states, the connectivity of the frontal lobes is enhanced. This phenomenon may be related to the complexity of emotion regulation and decision-making processes, as negative emotions typically require more cognitive resources to process, leading to a more significant role for the frontal lobes in emotional evaluation and response. Additionally, negative emotions exhibit similarities in connectivity patterns with neutral emotions, explaining why individuals may still experience some negative emotional memories or associations when faced with neutral stimuli (41). Therefore, the connectivity patterns observed in this study not only demonstrate the important role of the frontal and parietal lobes in emotional processing but also provide crucial insights into the neural mechanisms underlying emotional states. By further exploring the relationship between these dynamic connections and known emotional pathways, we can gain a deeper understanding of emotion recognition and its impact on behavior and mental states.

The significant increase in attention within specific brain regions when identifying specific emotional states provides important clues for understanding the neural basis of emotions. Attention weights are primarily concentrated in the frontal and temporal lobes, which aligns with findings from related research (42). Studies have shown that when individuals are confronted with emotional stimuli, the activity level of brain regions such as the frontal and temporal lobes significantly increases. The frontal lobe is believed to play a key role in higher cognitive functions and emotional regulation, while the temporal lobe is closely associated with the processing of emotions. Furthermore, relevant research consistently indicates a close relationship between the activity of these regions and individuals’ sensitivity to emotional information as well as the allocation of attention. This distribution of attention weights not only reveals the complexity of emotional processing but also emphasizes the division of labor and collaboration of the brain in emotion recognition.

By constructing probability distributions in a high-dimensional space from features extracted from the raw EEG signal, we captured the similarities between data points and transformed them into probability distributions in a low-dimensional space. After training with DGAT, the sample distribution became clearer, and the level of confusion among samples diminished. The clustering of different emotional states can provide guidance for model improvement. To further evaluate the performance of the proposed model, we calculated the confusion matrix of the DGAT model’s results, demonstrating its capability to accurately distinguish among different category data across the two datasets. The results of the ablation experiments validated that DGAT has an advantage in extracting emotion state-related features. It was observed that replacing the DGAT module with GAT significantly reduced the model’s emotional recognition performance. This underscores the ability of DGAT to dynamically learn the relationships between different channels and utilize dynamic adjacency matrices to lessen dependence on specific adjacency structures. Traditional static graph attention networks exhibit certain limitations in brain emotion classification tasks, relying on pre-defined fixed adjacency matrices during training. In contrast, the dynamic graph attention network enhances feature expressiveness and promotes a nuanced understanding of emotional states by dynamically learning inter-channel relationships. Through dynamic relationship learning, DGAT reduces reliance on specific adjacency structures, showcasing strong adaptability, enhanced expressiveness, and more precise emotion classification capabilities. When handling complex sequential data like EEG signals, DGAT effectively captures dynamic changes, improving the accuracy and practicality of emotion recognition.

## 5 Conclusions

We propose a dynamic graph attention network-based framework for EEG emotion recognition, referred to as DGAT. By utilizing dynamic adjacency matrices, DGAT dynamically learns the relationships between different channels, employing a multi-head attention mechanism to compute multiple attention heads in parallel. This approach reduces reliance on specific adjacency structures while enabling the model to learn information in different subspaces. Comparative experiments on the SEED and DEAP datasets against existing models demonstrate that in both subject-dependent and subject-independent experiments, the DGAT model, through dynamic adjacency matrices, overcomes the limitations of current graph neural network models that rely on pre-defined fixed adjacency matrices during training. This results in higher emotional classification accuracy, showcasing superior adaptability and feature expressiveness of the model. These findings provide valuable insights for the emotion recognition from EEG signals and subsequent research in emotion-based brain-computer interfaces. Additionally, our analysis of the experimental results revealed that, although the DGAT method exhibits commendable performance across the majority of testing scenarios, its efficacy in subject-independent experiments remains notably suboptimal. This observation has prompted us to undertake a more profound investigation, particularly concerning the applicability of the method and the potential challenges it may encounter in real-world implementations.

We recognize that individual differences among subjects could exert a significant influence on the performance of DGAT. These disparities may manifest not only in the data features but also in relation to various factors including each subject’s activity patterns, perceptual capabilities, and contextual environments. In light of this realization, we intend to explore advanced methodologies such as transfer learning and domain adaptation in our forthcoming research endeavors. These strategies are anticipated to offer novel perspectives for addressing the existing challenges. By enhancing the model’s adaptability within subject-independent contexts and augmenting its generalization capacity, we aim to broaden the scope of its applicability while maintaining performance integrity. Such an approach is anticipated to substantially elevate DGAT’s performance in subject-independent experiments and establish a robust foundation for the broader applicability of this method in practical scenarios.

## Data Availability

The original contributions presented in the study are included in the article/supplementary material. Further inquiries can be directed to the corresponding author.
